# Longitudinal impact of past-year reproductive coercion on contraceptive use dynamics in Sub-Saharan Africa: evidence from eight population-based cohorts

**DOI:** 10.1016/j.eclinm.2024.103056

**Published:** 2025-01-10

**Authors:** Shannon N. Wood, Haley L. Thomas, Georges Guiella, Rosine Mosso, Peter Gichangi, Simon P.S. Kibira, Fredrick Makumbi, Pierre Z. Akilimali, Funmilola M. OlaOlorun, Elizabeth Omoluabi, Michele R. Decker

**Affiliations:** a Department of Population, Family and Reproductive Health, Johns Hopkins Bloomberg School of Public Health, Baltimore, USA; b Bill & Melinda Gates Institute for Population and Reproductive Health, Department of Population, Family and Reproductive Health, Johns Hopkins Bloomberg School of Public Health, Baltimore, USA; c Institut Supérieur des Sciences de la Population (ISSP/University of Ouagadougou), Ouagadougou, Burkina Faso; d Ecole Nationale Superieure de Statistique et Appliquee d’Abidjan (ENSEA), Abidjan, Cote d’Ivoire; e Technical University of Mombasa, Mombasa, Kenya; f Department of Public Health and Primary Care, Faculty of Medicine and Health Sciences, Ghent University, Belgium; g Makerere University School of Public Health, Kampala, Uganda; h Kinshasa School of Public Health, Kinshasa, Democratic Republic of the Congo; i College of Medicine, University of Ibadan, Ibadan, Nigeria; j Faculty of Natural Sciences, University of the Western Cape, Cape Town, South Africa; k Johns Hopkins School of Nursing, Baltimore, USA

**Keywords:** Reproductive coercion, Contraception, Reproductive health, Sub-Saharan Africa, Longitudinal

## Abstract

**Background:**

Reproductive coercion (RC) is a type of abuse where a partner intentionally attempts to interfere with fertility through deception or violence, often by manipulating one’s contraceptive use or reproductive decision-making. Cross-sectional studies on the magnitude of RC across sub-Saharan Africa have noted associations with contraceptive use. No studies have longitudinally examined RC experiences as related to future contraceptive dynamics, including discontinuation or forgoing use altogether.

**Methods:**

Two rounds of longitudinal population-based cohorts across eight sites in sub-Saharan Africa, from November 2020 to January 2023, were used to prospectively examine past-year RC’s impact on future contraceptive dynamics (discontinuation and switching vs. continuation for contraceptive users at baseline; adoption vs. continued non-use for contraceptive non-users at baseline) using bivariate and multivariable multinomial and logistic regression.

**Findings:**

Minimal differences in women’s past-year RC experiences were observed over a two-year period. In many settings, RC prevalence decreased. Only in Uganda did past-year RC increase between rounds (15.8% to 17.8%). RC’s impact on contraceptive dynamics over one year differed by setting. In Burkina Faso, women with past-year RC had a three-fold increased risk of discontinuing contraception, as compared to continuing (RRR = 2.63; 95% CI = 1.28–5.42; p<0.01). In Uganda, past-year RC was marginally associated with reduced odds of contraceptive adoption, compared to continued non-use (p<0.1).

**Interpretation:**

In this first longitudinal study of RC, trajectories varied across settings, as did RC’s impact on contraception, affirming the importance of context. Future work should clarify RC trajectories and drivers thereof. Providers must be aware of RC leading to potential discontinuation. Contraceptive provision must be flexible and reflect women’s life circumstances, including partner dynamics.

**Funding:**

This work was supported, in whole, by the 10.13039/100000865Bill & Melinda Gates Foundation [INV-046501 and INV-009639]. Under the grant conditions of the Foundation, a Creative Commons Attribution 4.0 Generic License has already been assigned to the Author Accepted Manuscript version that might arise from this submission.

**Research in context**

**Evidence before this study**

Reproductive coercion (RC) is a type of abuse where a partner intentionally attempts to interfere with fertility through deception or violence, often by manipulating one’s contraceptive use or reproductive decision-making. We searched PubMed for the terms ((“reproductive coercion”) AND (contraception)) AND (“Africa South of the Sahara” [Mesh]) up to February 26, 2024, to identify studies exploring the relationship between RC and contraceptive use in sub-Saharan Africa. This search yielded 14 articles, eight of which did not explore contraceptive use as an outcome and three of which examined intimate partner violence as the exposure instead of RC. Three studies focused on RC as a primary exposure and found associated significant decreases in modern contraceptive use and significant increases in covert contraceptive use. Three qualitative studies, all conducted in Kenya, described the role of partners in contraceptive use decisions. Partner pressures against contraceptive use led women to use covertly or switch to more discrete methods upon discovery by their partner. No studies longitudinally examined RC’s impact on contraceptive dynamics.

**Added value of this study**

While identified cross-sectional and qualitative studies suggest RC is a threat to contraceptive use, the impact of RC experiences on contraceptive changes over time, including discontinuing, switching, or adopting contraception, has not been examined. In this study, we used two rounds of population-based cohorts to prospectively examine RC trajectories and contraceptive use dynamics in the subsequent year across eight sites in sub-Saharan Africa. We found that women in Burkina Faso who experienced RC in the year prior had nearly three-fold risk of discontinuing, as compared to continuing, contraceptive use by one year later. In Uganda, past-year RC experiences in the year prior were marginally associated with reduced odds of contraceptive adoption, compared to continued non-use, one year later.

**Implications of all the available evidence**

Patterns of contraceptive dynamics vary across settings and highlight a need for context-specific research and programming. Regardless, providers must be aware of potential RC and assist women in maximizing their reproductive preferences, even in the face of partner opposition.

## Introduction

Maternal mortality is unacceptably high and largely preventable, with an estimated 223 maternal deaths per 100,000 live births globally in 2020^1^; this burden is not shared equally, as 70% of maternal deaths globally occur in sub-Saharan Africa.^1^ Contraception has the potential to mitigate maternal mortality by preventing unwanted and/or high-risk pregnancies.^2^ Modeling approaches estimate that increased contraceptive use among those in need of contraception could prevent over 100,000 maternal deaths annually.^3^ Despite its benefits, contraceptive use remains low even for those wishing to avoid pregnancy.^4^ Commonly cited reasons for contraceptive nonuse include concerns about side effects or health risks,^5^, ^6^, ^7^ infrequent sex,^5^^,^^7^ or opposition from a loved one, such as a partner.^5^, ^6^, ^7^, ^8^

Partner opposition to contraception can hold severe repercussions—specifically, reproductive coercion (RC) is a type of abuse where a partner intentionally attempts to interfere with fertility through deception or violence.^9^ Known RC behaviors comprise three subtypes—pregnancy coercion (i.e., forcing someone to get pregnant), birth control sabotage (i.e., destroying contraception or preventing someone from accessing services), and abortion coercion (i.e., forcing someone to terminate a wanted pregnancy or keeping someone from getting a wanted abortion).^10^ Experiences of RC have been linked to a range of distal health outcomes, including unwanted pregnancy,^11^^,^^12^ sexually transmitted infections,^13^ and poor mental health.^14^, ^15^, ^16^ More proximally, RC is a threat to contraceptive use due to both direct and indirect contraceptive interference.^9^^,^^17^, ^18^, ^19^, ^20^

In sub-Saharan Africa, recent studies have quantified RC prevalence,^21^ identified associations between RC and contraceptive use cross-sectionally,^17^^,^^19^^,^^20^ and noted unique drivers of RC across culturally diverse settings.^19^^,^^21^ This research speaks to the difficulty women face when attempting to continue use of contraception, particularly if using their method covertly (i.e., without their partner’s knowledge).^18^^,^^22^ Type of method used (more concealable)^23^ and method effectiveness (more effective)^24^ have additionally been linked to RC in other settings, but not within sub-Saharan Africa.^17^ Moreover, no studies within sub-Saharan Africa have examined RC’s impact on contraceptive use over time—these longitudinal data are necessary to understand potential drivers in contraceptive dynamics for women trying to avoid pregnancy. For women who use contraception, RC could lead to contraceptive discontinuation should a partner interfere with use. Conversely, RC could impede women’s contraceptive use altogether by limiting their ability to adopt a contraceptive method. The present study utilizes two rounds of population-based cohorts to prospectively examine RC trajectories and the impact of past-year RC on contraceptive dynamics in the subsequent year (discontinuation and switching vs. continuation among contraceptive users; adoption vs. continued non-use among non-users) across eight sites in sub-Saharan Africa.

## Methods

### Overview of Performance Monitoring for Action

Data for this study come from Performance Monitoring for Action (PMA), a research platform that administers annual population-based surveys at the household, female, and facility levels in eight countries in sub-Saharan Africa and Asia.^25^ Using a multi-stage stratified clustered design with probability proportional to size sampling of enumeration areas, PMA obtains nationally or sub-nationally representative estimates of family planning indicators. Survey data are collected using mobile phones with the Open Data Kit (ODK) software by trained resident enumerators (REs) who administer the survey face-to-face. Additional details on PMA methodology, including the longitudinal study design, can be found at pmadata.org.

The present analyses utilize Phase 2 (P2) and Phase 3 (P3) female cross-sectional and longitudinal data from eight sites in sub-Saharan Africa (Table 1). Data were collected in Burkina Faso; Côte d’Ivoire; Kongo Central, Democratic Republic of Congo (DRC); Kinshasa, DRC; Kenya; Kano, Nigeria; Lagos, Nigeria; and Uganda. P2 and P3 data were collected approximately one year apart in each site (Table 1). These rounds were selected given their inclusion of RC items.

**Table 1:** Sample details, by site.

	National or sub-national sample	Phase 2 data collection	Phase 2 cross-sectional samples^a^ (n)	Phase 3 data collection	Phase 3 cross-sectional samples^a^ (n)	Phase 2-Phase 3 longitudinal samples^b^ (n)
Burkina Faso	National	Dec 2020–Mar 2021	2909	Dec 2021–Feb 2022	2906	3226
Côte d'Ivoire	National	Sep 2021–Dec 2021	1660	Sep 2022–Jan 2023	1692	1720
Kongo Central, DRC	Sub-national	Dec 2020–Feb 2021	877	Dec 2021–Apr 2022	863	716
Kinshasa, DRC	Sub-national	Dec 2020–Feb 2021	859	Dec 2021–Mar 2022	951	731
Kenya	National	Nov 2020–Jan 2021	4536	Nov 2021–Dec 2022	4804	4010
Kano, Nigeria	Sub-national	Dec 2020–Jan 2021	558	Dec 2021–Jan 2022	547	652
Lagos, Nigeria	Sub-national	Dec 2020–Jan 2021	626	Dec 2021–Jan 2022	632	585
Uganda	National	Oct 2021–Nov 2021	2041	Sept–Oct 2022	2238	1611

a Cross-sectional samples were restricted to partnered women in need of contraception. Women were categorized as “in need of contraception” at each phase if they reported being sexually active, not pregnant, fecund, not wanting any more children, or not wanting to have another child soon/right now.

b Longitudinal samples were limited to partnered women with complete RC data who participated in both Phase 2 and Phase 3.

### Analytic samples

Cross-Sectional Samples: Cross-sectional analyses include partnered women of reproductive age (15–49) who were in need of contraception at each time point (Table 1). Women were categorized as “in need of contraception” if they reported being sexually active, not currently pregnant, fecund, not wanting anymore children, or not wanting to have another child soon/right now, per time point.

Longitudinal Samples: Women of reproductive age (15–49) who participated in Phase 1 (P1) and were not lost to follow-up (LTFU) between P1 and P2 were eligible to complete the P2 female survey (Burkina Faso n = 5310; Côte d’Ivoire n = 3035; Kongo Central, DRC n = 1527; Kinshasa, DRC n = 1989; Kenya n = 6979; Kano, Nigeria n = 1000; Lagos, Nigeria n = 1108; Uganda n = 2974). In P3, these same women were eligible to complete the female survey; however, some were lost to follow-up between P2 and P3 (Burkina Faso LTFU n = 1002 [18.9%]; Côte d’Ivoire n = 635 [20.9%]; Kongo Central, DRC n = 431 [28.2%]; Kinshasa, DRC n = 385 [19.4%]; Kenya n = 883 [12.7%]; Kano, Nigeria n = 154 [15.4%]; Lagos, Nigeria n = 211 [19.0%]; Uganda n = 671 [22.6%]). Of the women in the longitudinal sample who were surveyed in both P2 and P3 (Burkina Faso n = 4308; Côte d’Ivoire n = 2400; Kongo Central, DRC n = 1096; Kinshasa, DRC n = 1604; Kenya n = 6096; Kano, Nigeria n = 846; Lagos, Nigeria n = 897; Uganda n = 2303), the final sample was limited to those who were partnered (married or cohabiting) and had complete RC data (Table 1). As a sensitivity analysis, samples were further limited to women in need to contraception (i.e., sexually active, not currently pregnant, fecund, not wanting anymore children, or not wanting to have another child soon/right now at P2; not shown).

### Measures

The primary exposure, past-year RC (binary), was measured in each phase via five items from the pregnancy coercion sub-scale of the RC Scale,^11^ reflecting both pregnancy coercion and birth control sabotage items. Items were previously modified for the sub-Saharan African context^26^ and validated within these study sites.^21^ Specifically, past-year RC was assessed as an affirmative response to any of the following behaviors by a husband or partner (yes/no response to each item): 1) Made you feel bad or treated you badly for wanting to use a family planning method to delay or prevent pregnancy; 2) Tried to force or pressure you to become pregnant; 3) Said he would leave you if you did not get pregnant; 4) Told you he would have a baby with someone else if you did not get pregnant; 5) Taken away your family planning or kept you from going to the clinic to get family planning. For longitudinal analysis, P2 data were used for examination of RC as a predictor of contraceptive dynamics.

Past-year contraceptive use was measured in P3 using the reproductive calendar. The calendar includes a retrospective report of all reproductive events (pregnancy, termination, birth) and contraceptive methods used, by month, starting at the most recent month and reporting backwards for 36 months. The number of months between the P2 and the P3 survey was calculated for each woman in the sample, and the calendar for those exact months was extracted from the full 36-month calendar, resulting in the P2–P3 reproductive calendar. Based on their calendar data, women were then classified as contraceptive users or non-users at P2.

Contraceptive users at P2 were then classified as continuous users, discontinuers, or switchers based on their contraceptive use between P2 and P3. Continuous users were women who reported using the same contraceptive method for every month between P2 and P3. Discontinuers were women who reported using a method at P2 but stopped using any method by P3. Women who became pregnant while using a method were also categorized as discontinuers. Switchers were women who reported using a method at P2 but switched to one or more different methods between P2 and P3. Notably, some women switched methods and then discontinued use; these women were categorized as discontinuers. Contraceptive dynamics were additionally examined as binary variables, with categorization as continuous users or not (discontinuer or switcher) and discontinuers or not (continuous user or switcher).

Contraceptive non-users at P2 were classified as continuous non-users or adopters based on their contraceptive use between P2 and P3. Continuous non-users were women who reported no method use between P2 and P3, including those who became pregnant and/or gave birth between P2 and P3. Adopters were women who were not using any method at P2 but began using at some month between P2 and P3.

Potential confounders were selected based on theory and prior research^21^^,^^27^ and include household-, relationship-, and individual-level characteristics, including residence (urban, rural), household wealth tertile (lowest, middle, highest), household composition (does or does not live with extended family), marital status (married, cohabiting), polygynous union, partner education (none or primary, secondary or higher), age (15–29, 30–39, 40–49), education (none, primary, secondary or higher), and parity (0–1, 2+).

### Statistical analysis

All analyses were conducted by site. First, cross-sectional samples for P2 and P3 were utilized to calculate the prevalence and 95% confidence intervals of RC, per timepoint; non-overlapping confidence intervals indicated significant differences between phases. Subsequent analyses focused exclusively on the longitudinal sample. The distribution of household-, relationship-, and individual-level characteristics were examined overall, by RC, and by contraceptive dynamics; design-based F statistics were utilized to examine significant differences in characteristics by RC and contraceptive dynamics, with characteristics with p<0.2 for both RC and contraceptive dynamics examined as potential confounders in subsequent models. Bivariate regression models were then used to examine the association between past-year RC and contraceptive dynamics outcomes. Specifically, among contraceptive users at P2, multinomial regression models examined risk of discontinuing or switching, compared to continued use. Among contraceptive non-users at P2, logistic regression models examined odds of contraceptive adoption compared to continued non-use. Correlates with p-value <0.2 from the bivariate models were assessed for collinearity; if the pairwise correlation was >0.4, the most conceptually relevant variable was selected for multivariable models. Multivariable models then examined these associations, adjusting for relevant confounders based on theory and confounding assessment, including residence, polygyny, partner’s education, and parity; adjusted models were only conducted in Burkina Faso, Côte d’Ivoire, Kenya, and Uganda, as RC samples were limited in sub-national sites. Variance inflation factors were estimated for all final models to check for multicollinearity. Sankey diagrams depicted RC experiences and contraceptive use dynamics (including method type) across the two survey rounds, by site.

All analyses were conducted in Stata 16 and were weighted to account for complex survey design and loss to follow-up to ensure the analytic samples remained nationally or sub-nationally representative. The weight for P2–P3 women was calculated as the P2 weight (for complex survey design), adjusted for the inverse of the predicted probability of having completed the P3 survey (LTFU). There was no differential LTFU in relation to RC, except in Kano, Nigeria, where women LTFU were less likely to report RC than those not LTFU (3.8% vs. 15.4%, p<0.001).

### Ethical protections

All respondents provided informed oral or written consent, per country guidelines. This study was approved by ethical review committees at Johns Hopkins Bloomberg School of Public Health; Comité d'Ethique pour la recherche en santé and the Ministère de la Santé et Ministère de l'Enseignement Supérieur, de la Recherche Scientifique et de l'Innovation in Burkina Faso; Comité d’Ethique de la Recherche Institut Pasteur de la Côte d’Ivoire; Comité d'Ethique de l'Ecole de Santé Publique de l'Université de Kinshasa in DRC; Kenyatta National Hospital-University of Nairobi (KNH-UON) Ethics Review Committee in Kenya; Lagos State University Teaching Hospital Health Research Ethical Committee and Kano State Health Research Ethics Committee, Aminu Kano Teaching Hospital Research Ethics Committee in Nigeria; and Makerere University School of Public Health Research and Ethics Committee in Uganda.

### Role of funding source

The funding source had no role in this manuscript.

## Results

### Cross-sectional samples

In P2, the majority of women were in need of contraception, ranging from 63.2% in Burkina Faso to 79.5% in Kenya (Table 2). Among women in need of contraception, past-year RC ranged from 5.3% in Lagos to 20.1% in Kongo Central; prevalence in five of eight sites was under 10%. In P3, the proportion of women in need of contraception ranged from 64.6% in Côte d’Ivoire to 80.5% in Kenya, and past-year RC ranged from 2.6% in Lagos to 16.9% in Uganda. While reports of past-year RC decreased from P2 to P3, differences were not statistically significant.

**Table 2:** Prevalence of past-year reproductive coercion among partnered women in cross-sectional samples, by site and by need for contraception.^a^

	Phase 2			Phase 3		
	In need of contraception % (n)	RC among women in need of contraception % (95% CI)	RC among women not in need of contraception % (95% CI)	In need of contraception % (n)	RC among women in need of contraception % (95% CI)	RC among women not in need of contraception % (95% CI)
Burkina Faso	63.2 (2909)	7.5 (6.0–9.3)	7.6 (5.8–9.8)	64.9 (2906)	4.6 (3.5–6.2)	4.7 (3.5–6.1)
Côte d'Ivoire	63.3 (1660)	6.7 (5.2–8.6)	9.1 (6.4–12.8)	64.6 (1692)	4.6 (3.1–6.9)	7.2 (4.6–11.0)
Kongo Central, DRC	70.1 (877)	20.1 (10.8–34.2)	15.7 (11.0–21.9)	71.1 (863)	10.5 (7.3–14.8)	9.7 (6.3–14.7)
Kinshasa, DRC	69.2 (859)	12.2 (8.2–17.9)	15.3 (11.2–20.6)	69.3 (951)	11.5 (7.9–16.6)	9.1 (5.9–13.8)
Kenya	79.5 (4536)	7.4 (6.2–8.9)	12.0 (9.0–15.7)	80.5 (4804)	6.3 (5.1–7.8)	14.4 (10.8–18.9)
Kano, Nigeria	67.0 (558)	7.2 (2.4–20.1)	4.1 (1.1–14.3)	68.2 (547)	3.1 (1.2–8.0)	3.5 (1.3–9.1)
Lagos, Nigeria	71.6 (626)	5.3 (3.6–7.7)	10.9 (6.9–16.8)	72.8 (632)	2.6 (1.4-4.6)	8.9 (4.5–17.0)
Uganda	73.2 (2042)	17.7 (14.8–20.9)	21.7 (17.7–26.2)	72.4 (2238)	16.9 (13.4–21.0)	20.2 (16.5–24.6)

Percentages are weighted.

a Women categorized as “in need of contraception” at each phase are those who reported being sexually active, not pregnant, fecund, not wanting any more children, or not wanting to have another child soon/right now.

### Longitudinal samples

The longitudinal samples were mostly comprised of younger women (aged 15–29) in Burkina Faso, Kongo Central, Kano, and Uganda, while women aged 30–39 were more common in Côte d’Ivoire, Kinshasa, Kenya, and Lagos (Table 3). Most women had at least a primary level of education in Kongo Central (89.8%), Kinshasa (99.7%), Kenya (94.4%), Lagos (98.3%), and Uganda (93.7%), whereas the majority of women in Burkina Faso (70.3%), Côte d’Ivoire (58.3%), and Kano (58.0%) had no formal education. Most partnered women in Kano (99.8%), Lagos (93.5%), Kenya (91.8%), Burkina Faso (91.4%), Côte d’Ivoire (71.5%), and Kinshasa (62.6%) were married, whereas women in Kongo Central (55.3%) and Uganda (55.2%) were primarily living with their partner outside of marriage. Polygyny varied substantially across sites but was most prevalent in Kano (43.6%) and Burkina Faso (43.0%) and least common in Lagos (10.2%) and Kinshasa (4.2%). The majority of women in all sites reported two or more live births. Most women in Burkina Faso, Côte d’Ivoire, Kinshasa, Kongo Central, Kano, and Uganda were not using contraceptives at P2, whereas the majority of women in Kenya and Lagos were using contraceptives at P2. Among those using contraceptives at P2, 1.5% (Kano) to 15.6% (Côte d’Ivoire) were doing so covertly.

**Table 3:** Characteristics^a^ of partnered women in longitudinal sample, by site.

	Burkina Faso (n = 3226)	Côte d'Ivoire (n = 1720)	Kongo Central, DRC (n = 716)	Kinshasa, DRC (n = 731)	Kenya (n = 4010)	Kano, Nigeria (n = 652)	Lagos, Nigeria (n = 585)	Uganda (n = 1611)
	Weighted %							
**Household**								
Residence								
Urban	16.2	53.1	–	100.0	24.1	31.0	100.0	21.9
Rural	83.8	46.9	100.0	–	75.9	69.0	–	78.1
Household Wealth Tertile								
Lowest	37.3	38.7	38.8	30.1	40.1	33.9	28.5	31.0
Middle	34.3	31.6	33.9	33.5	34.6	32.7	34.6	30.1
Highest	28.4	29.7	27.3	36.4	25.3	33.4	36.9	38.9
Household composition								
Does not live with extended family	58.8	52.5	72.5	58.9	73.8	84.3	76.2	63.4
Lives with extended family	41.2	47.5	27.5	41.1	26.2	15.7	23.8	36.6
**Relationship Dyad**								
Marital status								
Married	91.4	71.5	44.7	62.6	91.8	99.8	93.5	44.8
Living with partner	8.6	28.5	55.3	37.4	8.2	0.2	6.5	55.2
Polygynous union								
No	57.0	75.2	89.3	95.8	87.9	56.4	89.8	73.4
Yes	43.0	24.8	10.7	4.2	12.1	43.6	10.2	26.6
Partner education								
None/Primary	86.7	67.0	32.0	1.0	55.0	55.0	9.1	51.2
Secondary or Higher	13.3	33.0	68.0	99.0	45.0	45.0	90.9	48.8
**Individual**								
Age								
15–29	42.3	35.9	40.2	29.5	31.8	49.0	18.1	42.1
30–39	36.3	41.0	36.9	38.0	42.0	33.8	50.1	37.2
40–49	21.5	23.1	23.0	32.5	26.2	17.2	31.8	20.6
Education								
None	70.3	58.3	10.2	0.3	5.6	58.0	1.7	6.3
Primary	27.3	22.4	88.3	80.4	58.1	38.9	57.4	58.4
Secondary or Higher	2.4	19.2	1.6	19.4	36.3	3.1	40.8	35.4
**Reproductive**								
Parity								
0–1	16.0	18.0	15.7	21.1	12.7	13.3	16.9	15.7
2+	84.0	82.0	84.3	78.9	87.3	86.7	83.1	84.3
Contraceptive use								
No	67.2	73.8	64.6	51.4	36.8	86.8	47.4	56.9
Yes	32.8	26.2	35.4	48.6	63.2	13.2	52.6	43.1
Method mix^b^ among users								
LARC	54.9	26.6	39.6	27.3	41.3	31.4	21.8	37.9
Short-acting	31.8	40.6	16.9	10.8	44.0	51.7	16.8	33.7
Coital-dependent	5.6	7.5	25.2	21.7	2.4	9.4	45.6	9.5
Other/traditional	7.7	25.3	18.3	40.2	12.3	7.5	15.8	18.8
Covert use among users	13.3	15.6	10.4	8.4	6.5	1.5	10.7	13.2
RC (P2)	8.0	7.4	18.7	10.9	7.4	4.0	6.0	15.8
RC (P3)	4.3	4.8	9.1	9.9	7.1	2.9	4.2	17.8
Contraceptive dynamics between P2 & P3 among users at P2^c^								
Continuers	71.7	66.9	83.4	63.0	78.2	68.0	55.6	57.9
Discontinuers	23.3	25.8	11.4	25.6	12.1	18.0	18.5	26.0
Switchers	5.1	7.3	5.2	11.4	9.7	14.0	25.9	16.1
Contraceptive dynamics between P2 & P3 among nonusers at P2								
Continuous nonusers	82.4	85.6	81.6	77.4	69.3	88.4	78.4	71.7
Adopters	17.6	14.4	18.4	22.6	30.7	11.6	21.6	28.3

a Measured at Phase 2.

b LARC (IUD, implant); short-acting (oral contraceptive pill, injectable); coital-dependent (diaphragm, male condom, female condom, emergency contraception, foam/jelly, withdrawal); other/traditional (LAM, standard days, rhythm, male or female sterilization, other method).

c Women reporting male or female sterilization at P2 are not included in contraceptive dynamics analyses: Burkina Faso (n = 5), Côte d’Ivoire (n = 1), Kongo Central (n = 7), Kinshasa (n = 8), Kenya (n = 145), Kano (n = 0), Lagos (n = 4), Uganda (n = 39).

**Fig. 1:**
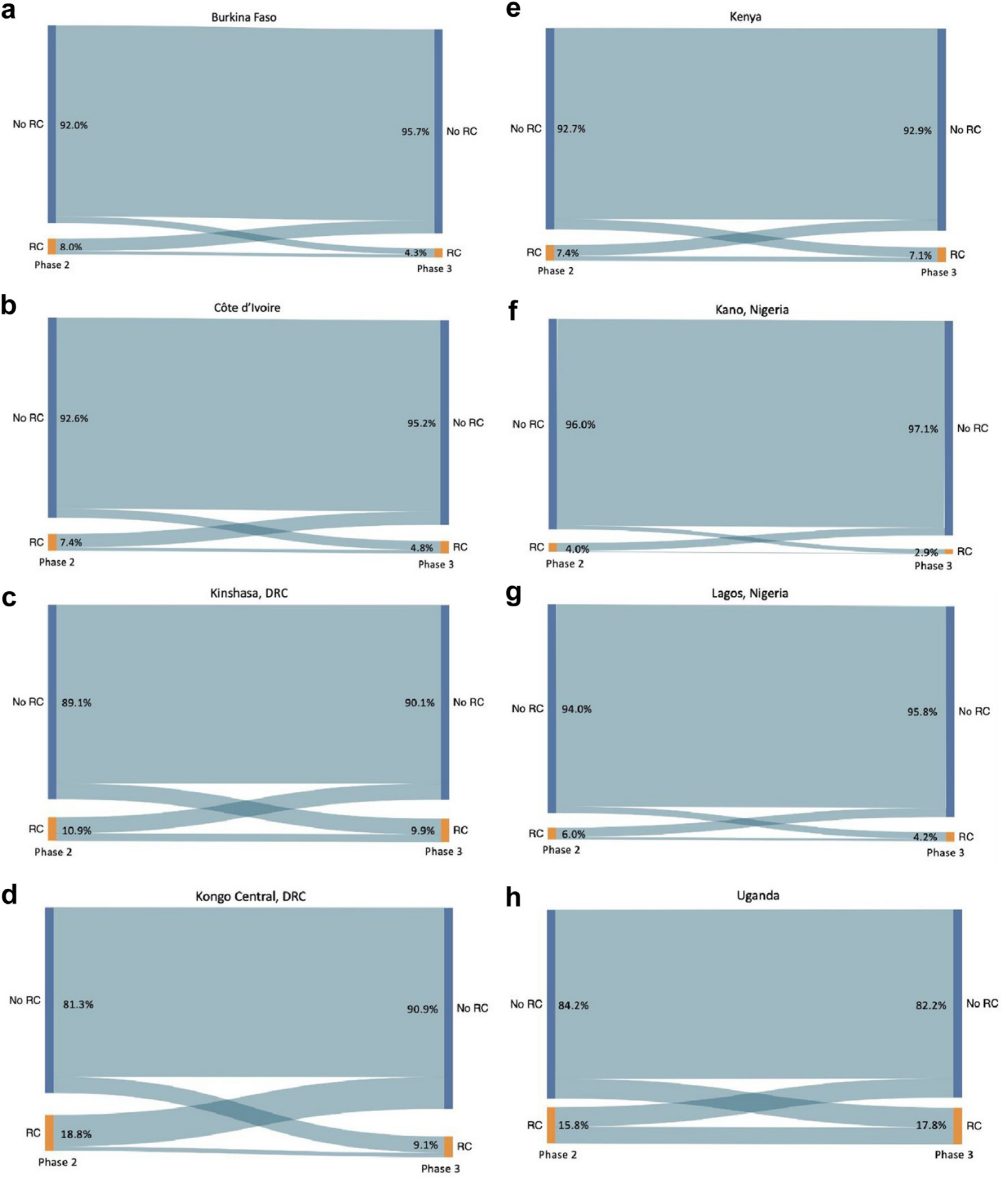
Reproductive coercion across timepoints, by site. Restricted to partnered women with complete survey data at both Phase 2 and Phase 3. The changes in RC between Phase 2 and Phase 3 were not statistically significant in any site except for Burkina Faso (8.0% in Phase 2–4.3% in Phase 3).

Among women followed longitudinally, prevalence of past-year RC reported in P2 ranged from 4.0% in Kano, Nigeria to 18.7% in Kongo Central, DRC (Table 3). Past-year RC decreased from P2 to P3 in every site except Uganda, with P3 reports ranging from 2.9% in Kano to 17.8% in Uganda (Fig. 1). The changes in RC between P2 and P3 were not statistically significant in any site except for Burkina Faso (8.0% in P2 to 4.3% in P3). Sensitivity analyses among women in need of contraception revealed similar trends within approximately one percentage point in either direction (data not shown).

**Fig. 2:**
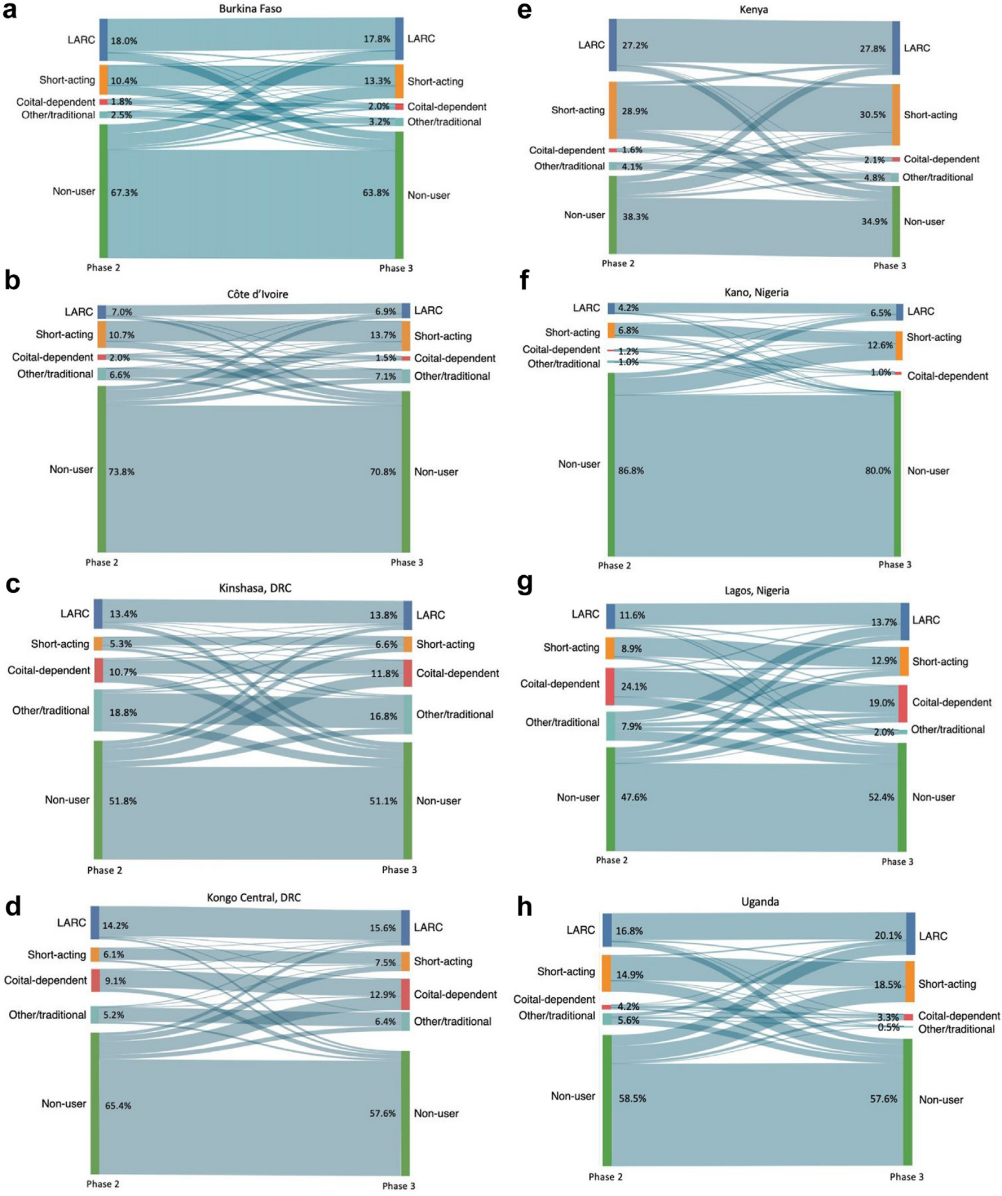
Contraceptive dynamics across timepoints, by site. Restricted to partnered women with complete survey data at both Phase 2 and Phase 3. LARC: IUD, implant; short-acting: injectable, pill; coital-dependent: male condoms, female condoms, diaphragm, emergency contraception, foam/jelly, withdrawal; other/traditional: rhythm, standard days, LAM, other traditional. IUD: intrauterine device; LARC: long-active reversible contraception; LAM: lactational amenorrhea method.

Contraceptive dynamics, including method mix, by site, are presented in Fig. 2. In P2, the majority of women in Burkina Faso, Côte d’Ivoire, Kongo Central, Kinshasa, Kano, and Uganda were contraceptive non-users; the overall proportion of non-users decreased in each of these sites by P3. The proportion of non-users from P2 to P3 increased in Lagos alone. Long-acting reversible contraceptives (LARCs) were the most common contraceptive method used in P2 and P3 in Burkina Faso, Kongo Central and Uganda, followed by short-acting methods in Côte d’Ivoire, Kenya, and Kano, other/traditional methods in Kinshasa, and coital-dependent methods in Lagos. Switching between methods or from use to nonuse, or vice versa, varied by site.

Among women using contraceptives at P2 (Table 4), RC prevalence among continued users ranged from 1.4% in Kano, Nigeria to 27.1% in Kongo Central, DRC; among discontinuers, RC prevalence ranged from 7.0% in Kenya to 14.0% in Uganda. In bivariate analysis, past year RC was associated with increased risk of contraceptive discontinuation in Burkina Faso only (discontinuation: 11.4%, continuation: 4.9%; p<0.05); this association remained significant in adjusted multinomial models (aRRR = 2.63; 95% CI = 1.28–5.42; p<0.01; Table 5).

**Table 4:** Bivariate analysis of past-year RC (at Phase 2) across contraceptive dynamics categories (between Phase 2-Phase 3), by site.

Contraceptive outcomes, among users at Phase 2	Burkina Faso			Côte d'Ivoire			Kongo Central, DRC			Kinshasa, DRC			Kenya			Kano, Nigeria			Lagos, Nigeria			Uganda		
	n = 1245			n = 441			n = 270			n = 346			n = 2348			n = 97			n = 303			n = 636		
	No RC %	RC %	RRR (95% CI)	No RC %	RC %	RRR (95% CI)	No RC %	RC %	RRR (95% CI)	No RC %	RC %	RRR (95% CI)	No RC %	RC %	RRR (95% CI)	No RC %	RC %	RRR (95% CI)	No RC %	RC %	RRR (95% CI)	No RC %	RC %	RRR (95% CI)
Continued use between P2–P3	95.1	4.9	ref	92.6	7.5	ref	72.9	27.1	ref	91.7	8.3	ref	93.1	6.9	ref	98.6	1.4	ref	95.8	4.2	ref	84.1	15.9	ref
Discontinuation between P2–P3	**88.6**	**11.4**	**2.50 (1.15–5.46)∗**	92.7	7.3	0.96 (0.39–2.35)	88.5	11.5	0.37 (0.08–1.76)	92.9	7.1	0.88 (0.39–2.00)	93.1	7.0	1.04 (0.61–1.78)	87.8	12.2	9.61 (0.43–214.05)	92.6	7.5	1.87 (0.55–6.35)	86.0	14.0	0.93 (0.51–1.68)
Switching between P2–P3	89.5	10.5	2.29 (0.71–7.43)	98.6	1.4	0.18 (0.02–1.58)	100.0	0.0	–	86.2	13.8	1.83 (0.43–7.82)	96.7	3.3	0.48 (0.18–1.23)	93.8	6.3	4.61 (0.24–90.02)	99.1	0.9	0.22 (0.03–1.61)	89.9	10.1	0.64 (0.25–1.62)

Contraceptive outcomes, among non-users at Phase 2	n = 1975			n = 1277			n = 438			n = 377			n = 1517			n = 555			n = 277			n = 936		
	No RC %	RC %	OR (95% CI)	No RC %	RC %	OR (95% CI)	No RC %	RC %	OR (95% CI)	No RC %	RC %	OR (95% CI)	No RC%	RC %	OR (95% CI)	No RC %	RC %	OR (95% CI)	No RC %	RC %	OR (95% CI)	No RC %	RC %	OR (95% CI)
Continued non-use between P2–P3	92.0	8.0	ref	92.4	7.6	ref	83.7	16.3	ref	88.1	11.9	ref	91.0	9.1	ref	95.8	4.2	ref	91.0	9.0	ref	80.7	19.3	ref
Adoption between P2–P3	88.9	11.1	1.43 (0.82–2.51)	93.1	6.9	0.90 (0.37–2.18)	82.6	17.4	1.08 (0.59–1.98)	82.3	17.7	1.60 (0.76–3.37)	91.1	8.9	0.98 (0.60–1.59)	97.6	2.4	0.56 (0.11–2.88)	94.3	5.7	0.61 (0.17–2.14)	**87.8**	**12.2**	**0.58 (0.31–1.07)**

Percentages are weighted.

Bolding indicates p<0.1, ∗p<0.05 from bivariate multinomial (users at P2) or logistic (non-users at P2) regression models.

**Table 5:** Multivariable analysis between past-year RC (Phase 2) and contraceptive use dynamics outcomes (Phase 2-Phase 3), by site.

Contraceptive outcomes, among users at Phase 2	Burkina Faso	Côte d'Ivoire	Kenya	Uganda
	n = 1245	n = 441	n = 2348	n = 636
	aRRR			
Continued use between P2–P3	ref	ref	ref	ref
Discontinuation between P2–P3	**2.63 (1.28–5.42)**	1.06 (0.41–2.71)	1.05 (0.61–1.80)	0.74 (0.41–1.34)
Switching between P2–P3	2.43 (0.77–7.72)	0.20 (0.02–1.73)	0.50 (0.19–1.30)	0.62 (0.24–1.58)

Contraceptive outcomes, among non-users at Phase 2	n = 1975	n = 1277	n = 1517	n = 936
	aOR			
Continued non-use between P2–P3	ref	ref	ref	ref
Adoption between P2–P3	1.50 (0.82–2.73)	0.97 (0.43–2.22)	1.03 (0.62–1.69)	0.62 (0.34–1.14)

Bolding indicates p<0.01 from multinomial (users at Phase 2) or logistic (non-users at Phase 2) regression models.

Adjusted for residence, polygyny, partner’s education, and parity.

Among women not using contraceptives at P2 (Table 4), RC prevalence among continued non-users ranged from 4.2% in Kano, Nigeria to 19.3% in Uganda; among adopters, RC prevalence ranged from 2.4% in Kano, Nigeria to 17.7% in Kinshasa, DRC. In bivariate models, past-year RC was marginally associated with a decreased odds of adoption in Uganda (continued non-use: 19.3%; adoption: 12.2%; p<0.1); these differences continued to trend towards significance in adjusted models (Table 5). There were no significant associations between past-year RC and continued non-use or adoption in adjusted logistic regression models (Table 5).

## Discussion

To our knowledge, this is the first longitudinal study of RC’s impact on contraceptive dynamics within sub-Saharan Africa. Surprisingly, there were minimal differences in women’s past-year RC experience over a two-year period. Patterns varied by site; in many settings, RC prevalence decreased between P2 and P3, and only in Uganda did past-year RC increase between rounds (15.8% P2 vs. 17.8% P3). Moreover, women in Burkina Faso who experienced RC in the year prior to P2 had nearly three-fold risk of discontinuing, as compared to continuing, contraceptive use by P3 (aRRR = 2.63; 95% CI = 1.28–5.42; p<0.01). In Uganda, past-year RC experiences in the year prior to P2 were marginally associated with reduced odds of contraceptive adoption, compared to continued non-use, by P3 (p<0.1). Results point to the potential role that RC can play in women’s ability to initially use contraception and continue its use, and the importance of context in understanding this relationship.

Overall, the relationship between past-year RC and contraceptive discontinuation was in the expected direction (i.e., increased discontinuation with recent RC experience). While results were excluded from further modeling due to small sample sizes, a trend towards increased discontinuation with RC experience was seen in both Kano and Lagos, Nigeria. Results were most pronounced in Burkina Faso, where 11% of contraceptive discontinuers, vs. only 4% of contraceptive continuers, experienced RC. These data provide evidence that RC experience plays a role in moving woman who are in need of contraception from use to non-use, and in turn, increasing their risk for both unintended pregnancy and maternal mortality. To protect women against RC’s adverse health impact, continued research must clarify the RC mechanisms and behaviors leading to discontinuation, including the potential role of method type and concealability. Burkina Faso is marked by the highest proportion of LARC users across all examined sites, despite RC’s links to increased discontinuation over a one-year period. High LARC use may be related to upward provider biases,^28^ as well as lack of information and/or barriers to LARC removal in Burkina Faso.^29^ Importantly, RC may be linked to distrust of specific methods of contraception,^30^ including longer-acting methods. Prior research in Uganda has additionally indicated doubled odds of contraceptive discontinuation within a one-year period if women did not discuss their contraceptive use with their partner prior to use (i.e., suggestive of covert use of contraception).^31^ While similar findings have not been replicated outside of Uganda, results on covert use and contraceptive discontinuation are instrumental to our interpretation of the present findings given the robust literature base linking RC to covert use in sub-Saharan Africa.^17^^,^^18^^,^^20^ In all study sites, except Kinshasa, Kenya, and Kano, greater than one in ten contraceptive users were doing so covertly, reflecting a potentially high-risk population for contraceptive discontinuation. Further research is needed to understand the cyclic mechanisms between RC and covert use and their relative contributions to contraceptive discontinuation, for women wishing to avert pregnancy.

Switching patterns were inconsistent across sites, and many settings were likely underpowered for such analyses, as there were few contraceptive switchers. To overcome power issues, sensitivity analyses were run to examine combined switching and discontinuation, however, such categorization does not elucidate coping mechanisms for women experiencing RC; for example, there is a major difference in women who experience RC and are able to switch to another, potentially more concealable method, vs. those who have to discontinue use entirely.^18^ Previous studies have indicated provider reluctance to allow women to switch methods, particularly when switching to a less effective method.^32^ These biases may be even more widespread for younger women, who have yet to initiate childbearing.^33^ Increased provider education on the importance of method satisfaction and the potential to switch methods should a woman dislike the method she is using is needed to maximize women’s safety and avoid unintended pregnancy.

Results surrounding RC and contraceptive adoption were also inconsistent. In Uganda, women who adopted a method, rather than continued not to use, were marginally less likely to experience RC (12.2% vs. 19.3%, respectively). While similar associations were not seen in other sites, the Ugandan findings are critical given that women experiencing RC and in need of contraception may forgo contraceptive use altogether, thus heightening risk for unintended pregnancy and its health sequelae. Notably, the present study only assessed RC in the year preceding contraceptive uptake—it is possible that women who have experienced previous RC or partner opposition were unlikely to try to use contraception again. Further, women have many reasons for choosing not to use contraception, despite not wanting to get pregnant, and contraceptive demand is not limited to partner interference/opposition. Fears surrounding hormonal contraception and its side effects remain pervasive throughout sub-Saharan Africa.^34^^,^^35^ Continued investment in contraceptive technologies that fit women’s preferences and minimize side effects (including when attempting to use covertly) is imperative to help women better manage their reproductive health and avoid pregnancy, if desired.

Results should be interpreted in light of several limitations. Foremost, sub-national sample sizes were limited, and as such, sub-national results were excluded from multivariable modeling. At both timepoints, RC was prevalent in sub-national settings of the DRC (Kongo Central and Kinshasa) and warrants further exploration with larger samples to better understand impact on contraceptive dynamics. In the Nigerian sites (Kano and Lagos States), in particular, small bivariate cells were seen for RC and continued use, limiting referent categories for further analysis. While national-level estimates are pivotal for power, national results may mask important within-context variation, as seen in substantial differences between sub-national sites in DRC and Nigeria. Further, RC measurement in sub-Saharan Africa is still in its infancy—while previously work has validated these items in sub-Saharan Africa specifically,^21^ the present measures likely do not reflect the full range of RC behaviors women may experience, including abortion coercion. Data surrounding abortion coercion in sub-Saharan Africa has been discussed qualitatively,^36^ however, quantitative measurement remains limited. Research on sensitive topics, including abortion, indicates that future research surrounding abortion coercion is feasible but must incorporate thoughtful study design, cultural sensitivity, and robust ethical safeguards to protect privacy given differing restrictions surrounding abortion and social desirability biases throughout sub-Saharan Africa. The present measures additionally exclude RC perpetrated by other parties, including family members, despite prior work describing their potential role in limiting women’s reproductive autonomy,^36^^,^^37^ likely leading to underestimates of RC and its impact. Lastly, this study only includes a one-year follow-up—while useful to illustrate changes, a true trajectory analysis would benefit from a cohort with longer follow-up duration.

Despite limitations, these results have several implications for family planning providers given the high proportions of contraceptive discontinuation and RC across several settings. Specifically, in Burkina Faso, Cote d’Ivoire, Kinshasa, DRC, and Uganda, approximately one in four women discontinued contraceptive use while still in need, increasing their risk for unintended pregnancy and maternal mortality.^2^^,^^3^ Moreover, withstanding Lagos, Nigeria, few women switched methods within the one-year period—prior literature points to the need to work with contraceptive providers to normalize switching, including due to reasons surrounding partner dynamics and interference.^32^^,^^33^ Training programs for contraceptive providers should emphasize the importance of initial conversations surrounding women’s needs and preferences to ensure that they adopt a method that fits their lifestyle, as well as flexibility should women need and/or want to switch methods if their initial method does not work for them. Given the fluidity of women’s life circumstances, including their partnerships, contraceptive provision must be flexible to meet women where they are.

Importantly, many women’s RC experiences were persistent over time; for these women, contraceptive use and/or continuation may be immensely difficult, as evidenced with increased discontinuation in Burkina Faso and marginal decreases in contraceptive adoption in Uganda. Innovative and adaptable reproductive safety strategies must be incorporated within family planning discussions to help women experiencing RC avoid unintended pregnancy.^18^ Ideas for provider assistance in concealing method use include cutting IUD strings, calling women to remind them of their return date for the injectable so that they do not need to bring scheduling cards home (if the woman indicates it is safe to call), and inquiring about safe places to hide contraceptive products during contraceptive counseling discussions.^18^ While norms change programs simultaneously work to prevent RC, provider recognition and counseling on covert use can help women avoid the repercussions of unintended pregnancy, while maximizing their own health and the health and well-being of their families.

## Contributors

Conceptualization–SNW, MRD. Project administration–GG, RM, PG, SPSK, FM, PZA, FMO, EO. Methodology–SNW, HLT, MRD. Formal analysis–HLT, SNW. Visualization–HLT. Data interpretation–SNW, HLT, MRD. Writing–original draft–SNW, HLT. Writing–review and editing, SNW, HLT, GG, RM, PG, SPSK, FM, PZA, FMO, EO, MRD. HLT and SNW accessed and verified the underlying data. All authors read and approved the final manuscript.

## Data sharing statement

Data are available upon request from https://www.pmadata.org/data/request-access-datasets.

## Declaration of interests

None.
